# An indolium inspired portable colorimetric sensor for cyanide recognition in environmental samples with smartphone integration†

**DOI:** 10.1039/d5ra00576k

**Published:** 2025-03-24

**Authors:** Kiran, Anju Ranolia, Priyanka, Anil Duhan, Rahul Kumar Dhaka, Snigdha Singh, Gaurav Joshi, Parvin Kumar, Devender Singh, Muhammad Wahajuddin, Jayant Sindhu

**Affiliations:** a Department of Chemistry, COBS&H, CCSHAU Hisar-125004 India; b Department of Chemistry, Delhi University Delhi-11007 India; c Department of Pharmaceutical Science, Hemvati Nandan Bahuguna Garhwal University Uttrakhand-244713 India; d Department of Chemistry, Kurukshetra University Kurukshetra Haryana-136119 India; e Department of Chemistry, Maharshi Dayanand University Rohtak-124001 India; f Institute of Cancer Therapeutics School of Pharmacy and Medical Sciences, University of Bradford UK jayantchem@gmail.com m.wahajuddin@bradford.ac.uk

## Abstract

Cyanide is a highly hazardous and fast-acting blood agent widely used in various industries, making its monitoring crucial due to its severe impact on living organisms. Considering this, we had developed a novel indolium based low-cost and portable sensor for the colorimetric and fluorogenic detection of cyanide ions. The probe displayed a distinct visual color change and selectively exhibits a fluorogenic “turn-off” response to cyanide ions in pure aqueous medium. The chemodosimetric-approach-based detection of cyanide ions has been confirmed using ^1^H NMR titration and DFT calculations. The presence of competing anions has minimal or no impact on the cyanide recognition process. The probe displayed an excellent detection limit as low as 3.78 nM with a response time of 5 s. The aqueous phase and optimum pH workability are some of the promising features of the developed probe. Additionally, a visual colorimetric strip based test was also developed using probe impregnated filter paper strips, which displayed a visual color change from red to colourless upon cyanide exposure. In addition to this, these test strips also showed excellent selectivity for cyanide ions, with no interference from fluoride or acetate ions. Furthermore, a smartphone-based protocol was developed to record the color change, demonstrating significant potential for cyanide detection without using sophisticated instrumentation. The formulation of the probe into an INHIBIT logic gate for facilitating cyanide recognition using electronic devices is one of the important applications.

## Abstract 2

A novel indolium based low-cost and portable sensor for the colorimetric and fluorogenic detection of cyanide ions.

## Graphical abstract

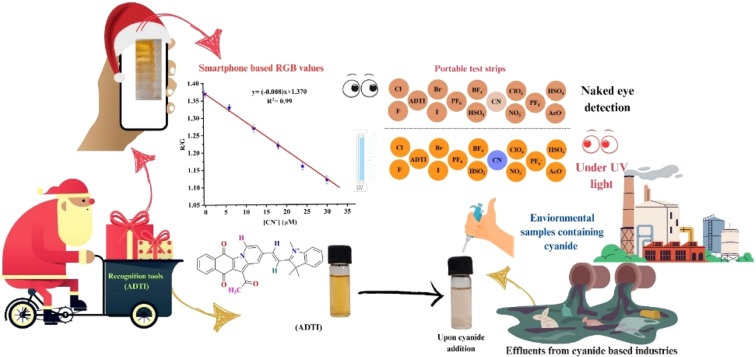

## Introduction

The harmful effects of cyanide, often referred to as “hydrocyanic acid” or “prussic acid” have been recognized for centuries.^1–4^ Its high volatility and low molecular weight make it very dispersive. Cyanides are utilized across the globe in numerous industrial applications including textile production, paint manufacturing, metal extraction, fertilizer production, coating processes, disinfectant production, organic synthesis, mining, photography and the pharmaceutical sector.^5–8^ Cyanide dependent industries produce 2–3 million tons of annual cyanide waste as reported by Towill *et al.*^9^ Additionally, cyanide is naturally produced in small amounts as cyanogenic glycosides by plants such as bitter almonds, coffee, chickpeas, apples, peaches, apricots, plums, cherries and other members of the Rosaceae family.^10–14^ The seeds of these foods contain a compound called amygdalin which upon hydrolysis produces HCN.^15^ Moreover, HCN gas is generated during the combustion of materials such as wool, silk and polyurethane. An average of 17.56–1553.98 μg of HCN was produced by cigarette smoke.^16^ Sodium nitroprusside, used to manage acute hypertensive crises, leads to the formation of cyanide. Rapid administration and prolonged use at high doses can result in toxic effects or kidney failure. Irreversible binding of a cyanide ion to the ferric ion (Fe^3+^) of cytochrome oxidase results into its deactivation, thus preventing oxygen exchange in the tissues, which ultimately leads to death.^17,18^ This interaction process is different from CO poisoning, as this leads to Hypoxia. Due to its harmful effects on human health and the environment, the real time monitoring of cyanide is crucial in both industrial and research settings, prompting ongoing efforts to establish new analytical strategies.^19–24^

The various analytical techniques such as voltammetry, ion chromatography, potentiometry, titrimetry and spectrophotometry are used for the determination of cyanide ion.^25–29^ While these methods fulfil legal requirements, many of these requires sophisticated equipment and are time-consuming, making them inappropriate for on-site detection. Thus, there is a crucial demand for development of rapid, inexpensive, selective and sensitive methodology for cyanide detection. Recently, colorimetric and fluorescence-based methodology has gained significant attention of scientific community in various fields such as environmental chemistry, biology and biochemistry,^30–34^ owing to their advantages, including on-site monitoring, low cost, ease of use, visual and rapid detection, high sensitivity and selectivity.^35–39^ Additionally, the field of fluorescence sensing has been expanded to a great extent with the development of compact and portable devices to meet the increasing demand for field applications.^40–46^

Indolium-based organic compounds, particularly indolium dyes, play a crucial role in cyanide detection due to their exceptional sensitivity and specificity.^47^ When exposed to cyanide ions, these compounds exhibit noticeable colorimetric or fluorometric shifts, making them highly effective for both visual and instrumental analysis.^48^ Other cyanide-detecting agents include Schiff bases, porphyrins, metal complexes (such as those incorporating copper or cobalt) and fluorescent probes like benzopyrylium salts and naphthalimides. These substances operate through diverse mechanisms, including nucleophilic addition, metal coordination and redox reactions, ensuring fast and precise cyanide detection in environmental and biological applications.^49–53^ However, some CN^−^ chemosensors may exhibit interference with other anions including F^−^, CH_3_COO^−^ and PO_4_^3−^ ions. The inability of organic chromophores to work in aqueous medium further restrict their use to lab based studies only. Hence, there is a strong need to develop selective and sensitive chemosensors which are operative in aqueous medium.

In our previous work, we have synthesized a novel sensor for cyanide ion with partial aqueous workability.^54,55^ Therefore, in continuation of our research work and in order to achieve aqueous phase workability, we had reported a novel naked eye and UV-light visible probe synthesized by the Knoevenagel condensation reaction (ADTI) for the real time monitoring of toxic cyanide ion in 100% aqueous medium. ADTI exhibits remarkable sensitivity and selectivity for CN^−^ ion in real samples with low LOD. Filter paper based test strips and smartphone based protocol were developed for on-site detection of cyanide ion.

## Experimental

### Materials and instrumentation

The organic building blocks including 2,3-dichloronaphthoquinone, pyridine, acetyl acetone, pyridine-4-carbaldehyde and indolium salt utilised in the present study were commercially procured from Sigma-Aldrich and used without further purification. Similarly, tetra-butylammonium salts for a range of anions, including cyanide, fluoride, chloride, iodide, nitrate, hydrogen sulfate, hexafluorophosphate, tetrafluoroborate, hydrogen sulphite and acetate were also purchased from Sigma-Aldrich. Both ^1^H and ^13^C NMR were recorded on Avance Neo 400 MHz NMR spectrometer in DMSO-*d*_6_. The FT-IR spectrometer from PerkinElmer (FL 6500) and Shimadzu LCMS-IT-TOF spectrometer were utilised for recording the infrared and mass spectra of the compound, respectively. All the absorption and emission studies were performed using Shimadzu UV-1900i and PerkinElmer 6500 spectrophotometer, respectively. The emission spectra of the compound were recorded at an excitation wavelength of 470 nm using a slit width and gain of 5 nm and 5, respectively. A test kit for the detection of cyanide was prepared using Whatman filter paper.

### Synthesis of 12-acetyl-6,11-dioxo-6,11-dihydrobenzo[*f*]pyrido[1,2-*a*]indole-2-carbaldehyde (ADC)

**Scheme 1:**
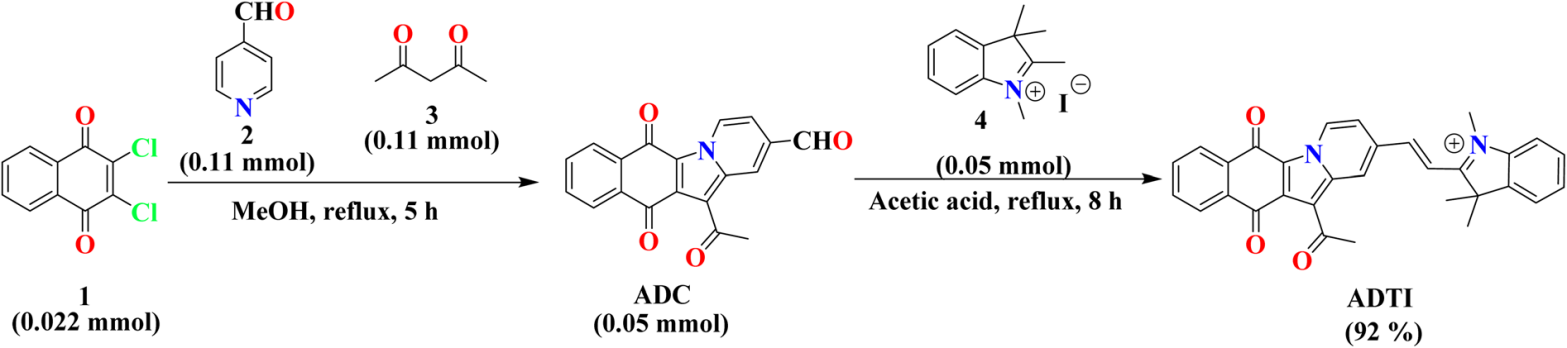
Acetic acid catalysed synthesis of probe (ADTI).

The reaction was attempted in a round bottomed flask (100 mL) containing a mixture of 2,3-dichloronaphthoquinone (1) (0.022 mmol), pyridine-4-carbaldehyde (2) (0.11 mmol) and acetyl acetone (3) (0.11 mmol) in EtOH (10 mL). The content of the flask was refluxed for 5 h in a pre-heated oil bath placed over magnetic stirrer. Thin layer chromatography was performed on pre-coated aluminium plates (TLC Merck) using EA : hexane (2 : 8, v/v) to record the reaction progress (Scheme 1). After complete consumption of reactants, the heating source was turned off and the reaction flask was clamped and suspended over an oil bath to allow it to attain ambient temperature. After cooling, the resulting solid was filtered, washed with ethanol and air dried to afford a red coloured solid (ADC) in 90% yield. The final composition of the compound was confirmed using mass spectrometry (*m*/*z* calculated 318.0758 [M + H]^+^; found 318.0763 [M + H]^+^).

### Synthetic protocol for (*E*)-2-(2-(12-acetyl-6,11-dioxo-6,11-dihydrobenzo[*f*]pyrido[1,2-*a*]indol-2-yl)vinyl)-1,3,3-trimethyl-3*H*-indol-1-ium iodide (ADTI)

To a stirred solution of ADC (0.05 mmol) in CH_3_COOH (15 mL) contained in a round bottomed flask (100 mL), 1,2,3,3-tetramethyl-3*H*-indol-1-ium iodide (4) (0.05 mmol) was added. The content of the flask was heated to reflux for 8 h. The reaction was monitored using TLC (DCM/MeOH (20 : 1, v/v)). During the course of the reaction, a solid product gradually separates out (Scheme 1). After complete consumption of the reactants, the reaction flask was kept at ambient temperature and the separated solid was filtered, washed with methanol and air dried to afford (*E*)-2-(2-(12-acetyl-6,11-dioxo-6,11-dihydrobenzo[*f*]pyrido[1,2-*a*]indol-2-yl)vinyl)-1,3,3-trimethyl-3*H*-indol-1-ium iodide (ADTI) in 92% yield.

### Spectral details

Red solid; yield: 92%; Mp: >300 °C; *R*_f_ (DCM/MeOH (20 : 1, v/v)) = 0.375; ^1^H NMR (400 MHz, DMSO-*d*_6_) *δ*_H_ 9.68 (d, *J* = 8 Hz, 1H), 8.91 (s, 1H), 8.55 (d, *J* = 16 Hz, 1H), 8.25 (dd, *J* = 8 & 2 Hz, 1H), 8.16 (m, 2H), 8.00–7.85 (m, 4H), 7.80 (d, *J* = 16 Hz, 1H), 7.68 (m, 2H), 4.24 (s, 3H), 2.81 (s, 3H), 1.84 (s, 6H); ^13^C NMR (100 MHz, DMSO-*d*_6_) *δ*_C_ 196.83, 182.30, 181.87, 175.11, 149.36, 144.56, 142.06, 137.31, 134.98, 134.62, 134.46, 133.95, 133.92, 129.87, 129.28, 127.78, 127.44, 126.83, 126.48, 123.45, 123.35, 121.82, 118.10, 116.32, 115.45, 99.98, 52.98, 35.46, 31.91, 25.28; LCMS: *m*/*z* calculated 473.1860 [M]^+^, found 473.1889 [M]^+^; elemental analysis: calculated C, 78.63; H, 5.32; N, 5.92; O, 10.14; found: C, 78.60; H, 5.31; N, 5.91; O, 10.12.

## Results and discussion

### Idea behind the design

**Fig. 1:**
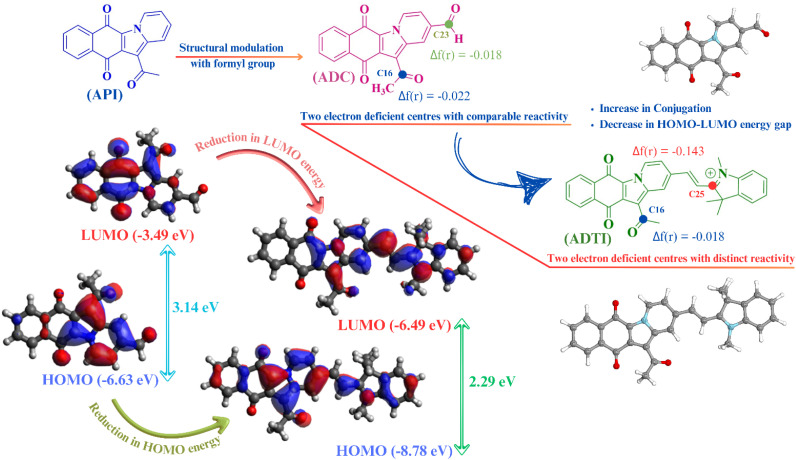
Structural modulation and localisation of local and global descriptors.

The rationale of the present work centres on the development of novel colorimetric and fluorometric probe with the potential to selectively and sensitively detect cyanide ions in the presence of various competing ions. Among different available strategies, the development of D–A framework with high degree of conjugation is one of the most recognised and widely accepted within the scientific community. The strategy for designing novel D–A framework began by structural modulation of 12-acetylbenzo[*f*]pyrido[1,2-*a*]indole-6,11-dione (API) with a formyl group resulting into 12-acetyl-6,11-dioxo-6,11-dihydrobenzo[*f*]pyrido[1,2-*a*]indole-2-carbaldehyde (ADC). The purpose of introducing formyl group is to extend the conjugation as well as to modulate the electrophilicity of the existing framework (API). The natural orbital analysis, performed using a DFT-based method, revealed the presence of two electrophilic centres (C_16_ and C_23_ atoms), with the higher electrophilicity of C_23_ over C_16_ being established through local reactivity descriptor (Fukui function). The high nucleophilicity of cyanide ion encouraged us to enhance the electrophilicity and regioselectivity of ADC. To achieve this, it was condensed with indolium unit to yield indolium based conjugated framework (ADTI). The condensation reaction resulted into a new electrophilic centre (C_25_) with enhanced electrophilicity, as established using Fukui function (Fig. 1). Moreover, a reduction in energy of FMO's with decreased HOMO–LUMO gap (2.29 eV) was noticed. This decrease in HOMO–LUMO gap resulted into a bathochromic shift in the absorption maxima of the compound. Within conjugated frameworks, chemodosimetric (reaction based approach) and H-abstraction strategies are widely utilised in cyanide ion recognition process. The reaction-based recognition of the cyanide ion involves a nucleophilic attack at the electrophilic site, resulting in the disruption of the ICT process thus leading to an observable variation in the spectral properties.

**Fig. 2:**
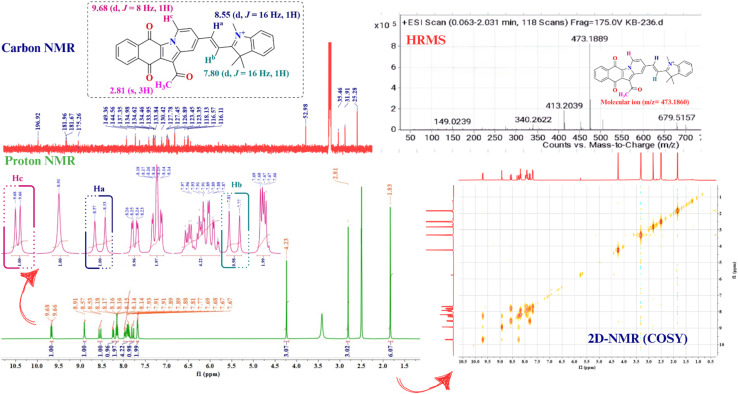
^1^H NMR, ^13^C NMR, COSY and mass spectra.

Considering all these facts, the designed compound (ADTI) has been synthesized *via* a two-step process and molecular geometry was established using various spectral techniques such as ^1^H, ^13^C NMR and mass spectrometry. The overall composition of ADTI was confirmed by mass spectra. After that the structure characterisation was performed using ^1^H and COSY NMR. The ^1^H NMR spectra of the compound exhibits two doublet at *δ*_H_ 8.55 and 7.80 ppm due to two protons with coupling constant 16 Hz which revealed the trans coupling of these protons (Fig. 2). The three proton attached to quaternized nitrogen resonates at *δ*_H_ 4.24 ppm as a singlet and the acetyl protons appeared at *δ*_H_ 2.81 ppm as singlet. The aromatic protons of the ring lie in the range of *δ*_H_ 9.68–7.68 ppm. Moreover, the peak at 196.83 ppm in ^13^C NMR spectra reflects the presence of carbonyl group in compound. The aromatic carbons resonate between 115–150 ppm and signals at *δ*_C_ 52.98, 35.46 and 31.91 ppm correspond to methyl groups.

### Photophysical studies

Donor–acceptor (D–π–A) framework based chromophores exhibit significant changes in their fluorescence spectra with change in solvent polarity.^56^ Due to its push–pull structure, the probe (ADTI) serve as a classical example of a D–π–A system. An increase in solvent polarity enhances the dipole–dipole interactions between the probe and solvent molecules, leading to a decrease in the energy of the excited state. The photophysical parameters of ADTI were recorded in solvents of different polarities at ambient temperature (Table S1†). The absorption spectra of ADTI displayed absorption maxima ranging between 476–503 nm (ESI Fig. S1†) which was assigned to the intramolecular charge transfer process. Similarly, its emission maxima were recorded from 572 to 641 nm in the same set of solvents (ESI Fig. S1†). Since light absorption is a very fast process, the absorption spectra of the probe were not affected significantly by solvent polarity.^57^ On the other hand, solvent polarity does have a significant effect on the excited state, as chromophores interact more with solvent molecules, resulting in larger dipole moments and a lowering of excited state energy. A significant variation in the emission spectra, accompanied by bathochromic shift of 69 nm, was noticed with increasing solvent polarity. A detailed insight of the solvatochromism behaviour of the designed probe can be gained by applying Lippert–Mataga equation on Stokes shift and solvent polarity function (Δ*f*). A positive linear correlation (*R*^2^ = 0.98) with solvent polarity was observed (ESI Fig. S2†). It is noteworthy to mention here that the excited state of ADTI is stabilised by polar solvent which further established that its emission arises from the ICT state.

### Naked eye recognition and sensing studies

**Fig. 3:**
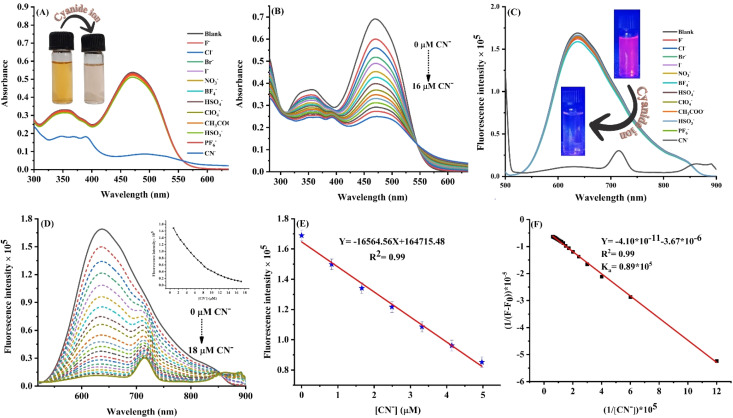
(A) Absorption spectra of the probe in the presence of different anions; (B) titration profile of ADTI with increasing concentration of cyanide ion (0–16 μM); (C) emission spectra of probe in the presence of various anions; (D) influence of cyanide concentration on the emission spectra of the probe; (E) LOD plot; and (F) Benesi–Hildebrand plot.

The selectivity of receptor towards a particular anion in a mixture of various competing anions is an important feature. Hence, the selectivity of ADTI towards cyanide ion was evaluated by visually examining the aqueous solution of probe in the presence of different anions. A visual color variation from yellow to colourless was observed only with CN^−^ ion, thus revealing the colorimetric selectivity of the developed probe towards cyanide ion (Fig. 3A). Additionally, the color change was also observed under UV illumination (365 nm) (ESI Fig. S3†). The red color of the aqueous solution of ADTI disappeared with the addition of CN^−^ ion, thus leading to a “turn-off” response (Fig. 3C). These outcomes finally establish the high selectivity of ADTI towards CN^−^ ion. Colorimetric responses in the field of sensing are always associated with absorption modulation. In the present study, a colorimetric response further directed us to perform detailed absorption based studies of ADTI with different anions. The effect of the presence of different analytes on the absorption spectra of ADTI probe was investigated using water as a medium. The purpose of selecting water as the medium for further recognition studies is to enhance the applicability of the developed probe in environmental monitoring and bio-sensing. The absorption maxima (470 nm) of probe remained unchanged in the presence of different anions except cyanide (Fig. 3A). The addition of cyanide ion reduced the absorption intensity of ADTI. These findings clearly demonstrate the strong selectivity of ADTI towards CN^−^ ions and align well with the results of the naked-eye detection experiment. The mode of interaction in a “ADTI-CN^−^” adduct was further established using absorption based titration experiment. A gradual addition of cyanide causes a consistent decrease in the intensity of absorption maxima due to the disruption of conjugation, which diminishes the ICT process (Fig. 3B). This type of colorimetric sensor offers robust and cost effective detection of cyanide ions without involving any sophisticated instrument.

The scope of the study was further extended by utilising emission based studies in cyanide recognition. The fluorescence response of ADTI was investigated in the presence and absence of anions using aqueous medium. Anions other than cyanide didn't influence the fluorescence emission of ADTI (Fig. 3C). However, the presence of cyanide ion resulted into a decrease in emission intensity with hypsochromic shift. The behaviour was likely observed due to the addition of cyanide ions, which disrupts conjugation, thereby producing a “turn-off” response. A gradual decrease in the emission intensity of ADTI with the incremental addition of cyanide ions to the solution was attributed to a complexation reaction (Fig. 3D). This variation in the fluorescence spectra of probe indicates the effective disruption of conjugation due to nucleophilic addition of cyanide ion, which ultimately obstructs the intramolecular charge transfer transition. The limit of detection (LOD) for cyanide recognition was calculated to be 3.78 nM, which is below the level recommended by the WHO (Fig. 3E).^48^ The binding constant calculated for this complexation reaction using titration studies was found to be 8.9 × 10^4^ M^−1^ (Fig. 3F). On the basis of the values of binding constant and detection limit, it has been concluded that the probe has the capability to recognise cyanide ion in environmental samples.

**Fig. 4:**
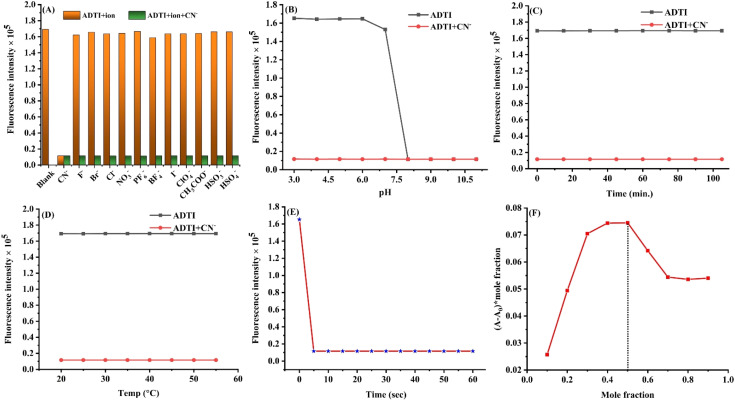
(A) Interference studies in the presence of other ion; (B) effect of pH on ADTI and “ADTI-CN^−^” adduct (C) response time; (D) photo-stability of ADTI and “ADTI-CN^−^” adduct; (E) thermal stability of ADTI and “ADTI-CN^−^” adduct; (F) Job's plot.

Further, to determine the selectivity of ADTI, the response of probe towards cyanide ion in the presence of other competing ions was investigated (Fig. 4A). It has been observed that the cyanide recognition ability of ADTI remained unaffected even in the presence of other competing ions. These outcomes reflect the highly selective nature of the probe.

Various environmental factors, such as pH, photostability and temperature play a significant role, as they can interfere with the recognition process and impact the overall sensing performance of the probe. Therefore, the influence of pH on the recognition potential of ADTI was explored (Fig. 4B). The outcome of this study revealed that the developed sensor has the capability to detect the CN^−^ ion within the pH range of 3–7. These outcomes reflect the potential ability of the probe to work under optimum pH range. In the basic pH, hydroxide ion competes with cyanide ion. After successfully establishing the workability of probe in optimum pH range, the photo stability of the probe and “probe-CN^−^” adduct was also assessed by irradiating their solution with UV light (254 nm) (Fig. 4C). The outcomes of the experiment revealed that the probe and “probe-CN^−^” adduct didn't exhibits photo-bleaching upon exposure to UV-irradiation. Similarly, the thermal stability for both was also assessed. It has been observed that the emission characteristic of both probe and “probe-CN^−^” adduct remained unaltered even at temperature greater than 50 °C. These findings revealed the high thermal stability of the probe and probe-CN^−^ adduct (Fig. 4D).

One of the major limitations of the already reported cyanide responsive probes is their inability to respond quickly to a μM amount of cyanide present in the solution. The response time of the probe towards CN^−^ ion was also evaluated by performing a time dependent study. It has been found that the probe displayed quick response within 5 s when exposed to CN^−^ ion (Fig. 4E). Moreover, the solution color changed from yellow to colourless immediately. Further, the stoichiometry of the probe to CN^−^ ion was determined using Job's continuous variation method (Fig. 4F). The obtained graph displayed the maximum value at mole fraction of 0.5, revealing a 1 : 1 binding stoichiometry of receptor to cyanide ion.

### Plausible sensing mechanism

**Fig. 5:**
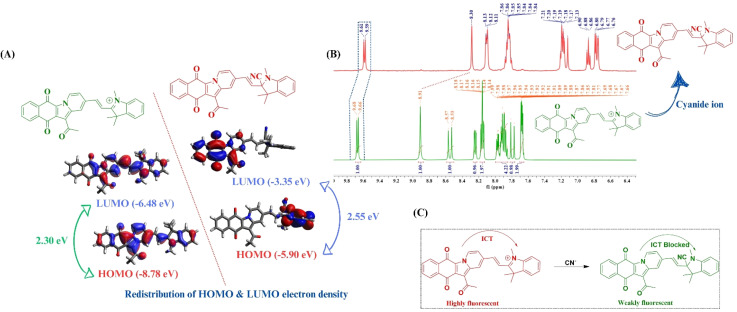
(A) DFT studies; (B) ^1^H NMR titration studies of ADTI in the presence of cyanide ion in DMSO-*d*_6_ and (C) plausible mode of interaction between ADTI and cyanide ion.

The binding stoichiometry of the probe with the cyanide ion was confirmed using Job's plot. To gain a deeper insight into the probable mechanism of interaction and to support the experimental evidence from photophysical studies, density functional theory (DFT)-based calculations were performed for the probe and the “probe-CN^−^” adduct. The ground state optimized geometry of ADTI revealed a planarity in the molecular structure thus facilitating the intramolecular charge transfer process. However, in the case of the probe-CN^−^ adduct, the molecule loses its planarity due to the disruption of conjugation upon the nucleophilic addition of the cyanide ion. Additionally, a significant redistribution of electron density was observed, which accompanies an increase in the HOMO–LUMO gap (Fig. 5A). The results of DFT studies are in strong corroboration with the experimental findings and supports the disruption of conjugation with the nucleophilic addition of cyanide ion.

Furthermore, to establish the nucleophilic addition of cyanide ion to C=N^+^ of the indolium moiety, ^1^H NMR titration was conducted in DMSO *d*_6_. In the ^1^H NMR spectra of ADTI, the two vinyl protons resonate as doublets at *δ*_H_ 8.55 and 7.80 ppm with a coupling constant 16 Hz. Upon cyanide addition, an upfield shift is observed in the NMR signals of these protons. Moreover, the signal corresponding to the aromatic protons also shifts to the upfield region. Based on the literature studies, the probable mechanism of sensing can be depicted as shown in Fig. 5B. From these findings, it can be concluded that the nucleophilic attack of the cyanide ion on the C=N^+^ group of the indolium moiety results in the disruption of conjugation, thereby inhibiting the flow of electrons from the donor to the acceptor part, resulting into a “turn-off” fluorescence response (Fig. 5C). Such behavior is commonly associated with the quenching of absorption and emission intensities.

### Real time application

The rapid growth of industrial activities, coupled with inadequate waste disposal practices, significantly contributes to environmental degradation. Cyanide is commonly found as a contaminant in wastewaters from various industries including metal cleaning, plating, electroplating, metal processing, automobile parts manufacture, steel tempering, mining, photography, pharmaceuticals, coal coking, ore leaching and plastics. Therefore, there is an urgent need to develop an effective analytical strategy for qualitative and quantitative estimation of cyanide ion in environmental samples. In order to demonstrate the practical utility of ADTI in cyanide recognition, real sample analysis was performed using tap water, Ganga river water and drinking water as different environmental matrices. The water samples are spiked with known concentration of cyanide ion and analysed using fluorescence technique. As can be seen from Table 1, the probe has the potential to detect the CN^−^ ion with excellent % detection (93.3–98.3%). Moreover, the developed method has been validated with the previously reported AgNO_3_ method.^58^ These findings revealed highly sensitive nature of probe, indicating its reliability and feasibility in cyanide recognition process.

**Table 1:** Real water sample analysis

Samples	[CN^−^] (μM)		Detection (%)
	Spiked	Detected	
River water (Ganga)	0	ND	
River water (Ganga)	3	2.80	93.3
Tap water	0	ND	
Tap water	3	2.95	98.3
Drinking water	0	ND	
Drinking water	3	2.89	96.3
AgNO_3_ method	3	2.88	96.0

### On site recognition studies

**Fig. 6:**
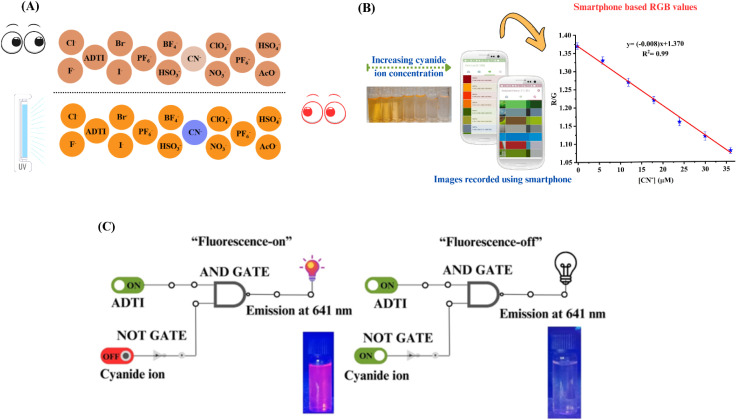
(A) Filter paper based test strip for cyanide detection under naked eye and UV-lamp; (B) smartphone based sensing of CN^−^ in aqueous solution of sensor ADTI; (C) INHIBIT logic gate.

To determine the practical application of the developed colorimetric and fluorometric probe for on-site detection of CN^−^ ions, the probe was impregnated onto a filter paper-based test strip. This approach facilitates easy and robust recognition without utilising sophisticated instrumentation.^54,55^ Initially, the strips were dipped into an ADTI solution (10^−3^ M) for impregnation. The solvent was then removed by vacuum drying the strips for 24 h. The dried strips, when visualized under visible and UV light, exhibited red and orange colors, respectively (Fig. 6A). Then, the strips were exposed to the aqueous solution of various analytes (anions). A discernible color change from red to colorless under visible light, and from orange to blue under UV light, was observed only with cyanide exposure. Fig. S4† represents the pictorial representation of the cyanide detection test strip. These outcomes demonstrated the practical applicability of the developed probe for on-site cyanide detection using ADTI-impregnated paper strips, thus extending the overall scope of the probe to remote areas.

In addition to probe impregnated paper strips, smartphone based monitoring of immediate color change is also one of the cheap and robust method for on-site detection.^58^ In the present study, the immediate color change of a ADTI solution upon cyanide addition has been utilised for the development of smart phone based recognition process. In this experiment, the ADTI solution (10^−5^ M) was mixed with five different known concentrations of CN^−^ in sample vials, and all the solutions were placed in containers lined up next to a blank sample. A photograph was captured using smartphone camera (Samsung Galaxy A32) positioned approximately 50 centimetres from a white background. The digital image analysis provided RGB (Red, Green, and Blue) data, with values ranging from 0 to 255 for each channel. For example, a completely black color would yield RGB values of 0, 0, 0, while pure white is associated with values of 255, 255, 255.

The “Colour Assist App” a free android application, was used to monitor RGB values in real samples, significantly reducing the analysis time as compared to other commonly used methods for determining RGB values (such as Image J, Matlab and Adobe Photoshop) (Fig. 6B). This app was used to analyze the glass vials containing only ADTI and those with varying known concentrations of CN^−^ ion. Relying solely on visual observation is not sufficient for detailed analysis, so a calibration curve was established with RGB values at different CN^−^ concentrations. The resulting plot displayed a linear relationship with an *R*^2^ = 0.99 for the R/G ratio against [CN^−^]. The fabrication of sensors for the development of electronic devices is fundamentally based on logic gates, which serve as the core building blocks for processing and interpreting data. These sensors can be integrated into complex systems to enable automation, control, and decision-making processes, making them crucial for advancements in the field of environmental recognition and bio-sensing.^59^ In the present study, the sensing behaviour of ADTI towards cyanide ion was also analysed using logic gates. The “turn off” fluorescence response of ADTI can be demonstrated by a molecular “INHIBIT” logic gate, considering ADTI and cyanide ion as two inputs and signal at 641 nm as output. The truth table has been represented in ESI Table S2.† In this case, the emission intensity of ADTI was high in the absence of cyanide ions, corresponding to the “ON” state with a logic value of “1” (Fig. 6C). The addition of cyanide ions to the solution led to a decrease in emission intensity, demonstrating the “OFF” state, which corresponds to a logic value of “0”.

### Comparison of the developed probe with previously reported probe

The performance of the synthesized probe has been compared with already available cyanide sensor. The probe displayed a distinct visual color change and selectively exhibit a fluorogenic “turn-off” response to cyanide ion. The presence of competing anions has minimal or no impact on cyanide recognition process. The probe displayed an excellent detection limit as low as 3.78 nM. The aqueous phase and optimum pH workability are some of the promising features of the developed probe. The probe displayed rapid response within 5 s. Generally, two approaches have been utilised in the detection of cyanide ion such as H-abstraction and chemodosimetric approach. The chemodosimetric approach based chemosensor exhibits higher selectivity and sensitivity for the cyanide ion as this approach involves chemical bond formation between the probe and cyanide ion which is highly reliable. Table S3† summarises the comparison of various analytical attributes of already reported and our developed probe. From the Table S3,† it has been concluded that the present probe displayed superior LOD than the already available probe.

## Current challenges and future prospects

Cyanide sensing is a critical area of research due to the compound's high toxicity and widespread use in industrial processes, posing significant environmental and health hazards. Current challenges in cyanide sensing include achieving high selectivity and sensitivity in complex environmental matrices, developing portable and cost-effective sensors for real-time monitoring, and for ensuring robust performance under diverse environmental conditions. Additionally, addressing the interference caused by other anions, broad pH applicability, low photo-stability and improving the response time of detection methods remain key obstacles. However, future prospects in this field are promising, with advancements in nanotechnology, material science and fluorescence-based techniques paving the way for highly efficient sensing platforms. Innovations such as colorimetric sensors array, multifunctional materials and integration with Internet of Things (IoT) systems could revolutionize cyanide monitoring, enhancing environmental safety and public health protection.

## Conclusion

In summary, a novel colorimetric and “turn off” fluorescent probe (ADTI) has been developed by structural modulations with extension of conjugation. The designed molecule displayed solvatochromism, excellent selectivity and sensitivity towards CN^−^ ion over other anions in aqueous medium. The LOD and binding constant was found to be 3.78 nM and 8.9 × 10^4^ M^−1^, respectively. The recognition mechanism involved the nucleophilic attack of cyanide ion and resulted into the disruption of ICT. The interaction mechanism has been established using DFT and ^1^H NMR studies. The designed probe has the potential to detect cyanide ion in environmental samples. Additionally, test strip impregnated with ADTI demonstrates its practical utility in the on-site monitoring of cyanide ion. Smartphone application based strategy also displayed its potential in cyanide recognition without using sophisticated instrumentation.

## Data availability

Data will be made available on request.

## Author contributions

Kiran – methodology, investigation, formal analysis, data curation, visualisation, writing – original draft; Anju Ranolia – writing – reviewing and editing; Priyanka – writing – reviewing and editing; Anil Duhan – reviewing and editing; Rahul Kumar Dhaka – reviewing and editing; Snigdha Singh – reviewing and editing; Gaurav Joshi – writing – reviewing and editing; Parvin Kumar – reviewing and editing; Devender Singh – reviewing and editing; Muhammad Wahajuddin – conceptualization, resources, reviewing and editing; Jayant Sindhu – supervision, software, conceptualization, project administration, writing – original draft.

## Conflicts of interest

The authors declare that they have no known competing financial interests.

## Supplementary Material

RA-015-D5RA00576K-s001
