# Supplementary material for: An indolium inspired portable colorimetric sensor for cyanide recognition in environmental samples with smartphone integration
Source: RSC Adv. 2025 Mar 24;15(12):9129–40. doi: 10.1039/d5ra00576k (PMC11932618; doi:10.1039/d5ra00576k)
Supplement: RA-015-D5RA00576K-s001 [file RA-015-D5RA00576K-s001.pdf]

Supplementary Information (SI) for RSC Advances.
This journal is © The Royal Society of Chemistry 2025

Supporting information

Indolium inspired portable colorimetric sensor for cyanide recognition in environmental samples with smartphone integration

Kiran[a], Anju Ranolia[a], Priyanka[a], Anil Duhan[a], Rahul Kumar Dhaka[a], Snigdha Singh[b], Gaurav Joshi[c,f], Parvin Kumar[d], Devender Singh[e], Muhammad Wahajuddin*[f], Jayant Sindhu*[a]

[a]Department of Chemistry, COBS&H, CCSHAU, Hisar-125004

[b]Department of Chemistry, Delhi University, Delhi-11007, India

[c]Department of Pharmaceutical Science, Hemvati Nandan Bahuguna Garhwal University, Uttrakhand-244713, India

[d]Department of Chemistry, Kurukshetra University, Kurukshetra, Haryana-136119, India

[e]Department of Chemistry, Maharshi Dayanand University, Rohtak-124001, India

[f] Institute of Cancer Therapeutics School of Pharmacy and Medical Sciences, University of Bradford, United Kingdom

Email- jayantchem@gmail.com; m.wahajuddin@bradford.ac.uk.

Contents

Supporting information 1
Experimental 2
UV–vis and fluorescence spectral measurement 2
Table of contents
Computational studies..2
Limit of detection calculation 3
^{1}H, ^{13}C NMR and Mass spectra of ADTI..3
UV-visible absorption and fluorescence spectra of ADTI in different solvents 5
Lippert Mataga plot..5
Visible changes in probe solution under naked eye and UV-lamp in the presence of analytes..6
Photophysical attributes of ADTI..6
Comparison of developed probe with previously reported probe..7
References..8

Experimental

UV–vis and fluorescence spectral measurement

Initially, the stock solution of 10^{-3} M of the synthesized probe was prepared in DMSO which was further diluted to 20 μM for further analysis. The solution of **ADTI** (20 μM) required for spectroscopic and colorimetric experiments was prepared in H_2O. All the titration experiments were performed at room temperature. 0.1 M Stock solution of tetrabutylammonium salt of various analyte such as F^-, Cl^-, Br^-, CN^-, HSO_4^-, ClO_4^-, NO_3^-, BF_4^-, PF_6^-, CH_3COO^-, HSO_3^- and I^- in water was used for sensing applications. The UV-vis absorption and fluorescence spectral profile were recorded by performing the experiment in triplicate.

Computational studies

The mechanism of cyanide sensing was theoretically evaluated using Density functional theory (DFT) calculations. All the calculations were performed on Tyrone workstation with Gaussian 16 software using B3LYP/6-311G (d,p) level of theory. The value of the Fukui functions was calculated using the following equation

$$f_i^+ = q^i(N+1) - q^i(N)$$

$$f_i^- = q^i(N) - q^i(N-1)$$

$$f_i^0 = (q^i(N+1) - q^i(N-1))/2$$

The atomic charge on i^{th} atomic site is denoted by the letter q^i. NBO analysis was used to establish each atom's inherent charge. The sites for electrophilic and nucleophilic attack on molecules are distinguished as

$$\Delta f(r) = f_i^+ - f_i^-$$

Atomic sites with $f(r) > 0$ are vulnerable to electrophilic attack, whereas sites with $f(r) < 0$ are more vulnerable to nucleophilic attack.

Limit of detection calculation

Limit of detection (LOD) was calculated based on the standard deviation (SD) of the blank and slope of the calibration curve at the level of approximation. The LOD was calculated according to the formula:

$$LOD = 3.3\ (SD/\sigma)$$

Where, SD stands for the standard deviation of blank and σ represents the slope of the calibration curve.

^{1}H, ^{13}C NMR and Mass spectra of ADTI

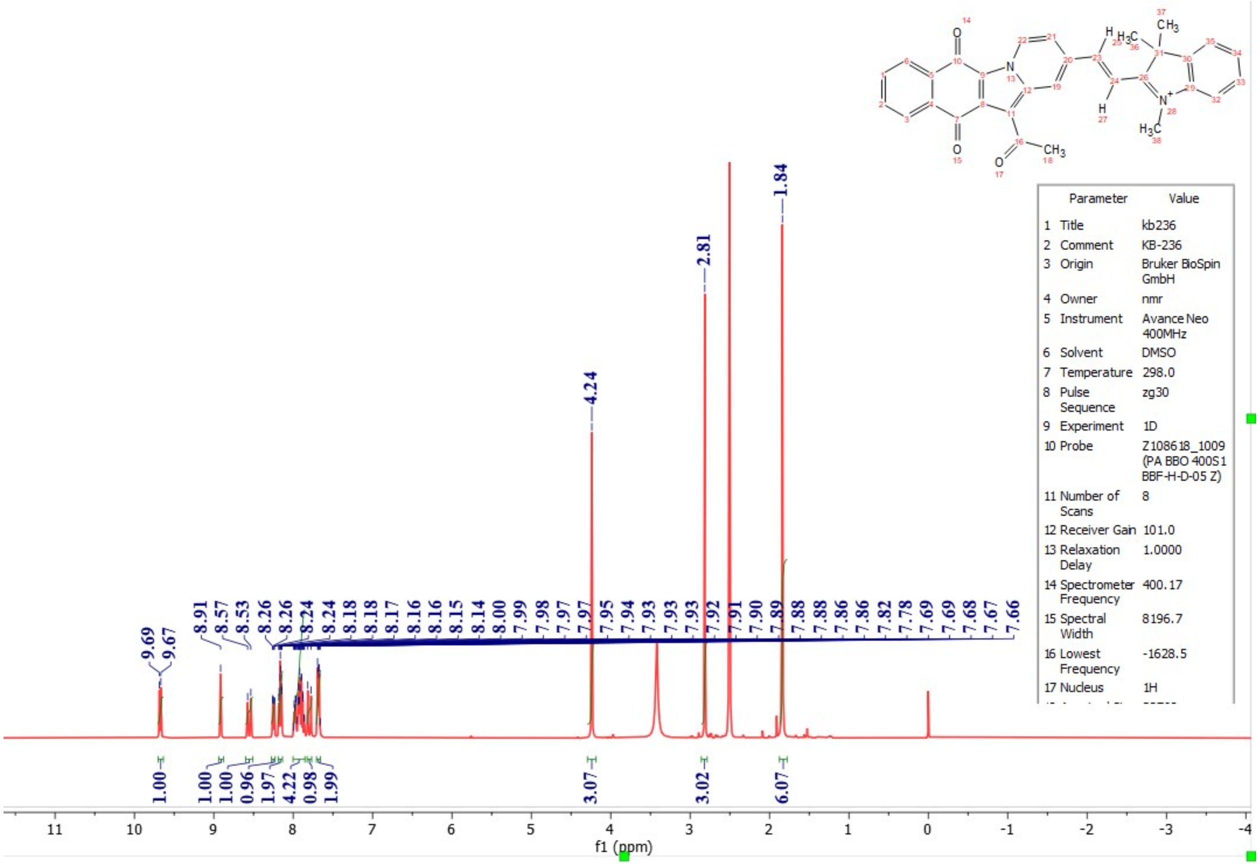

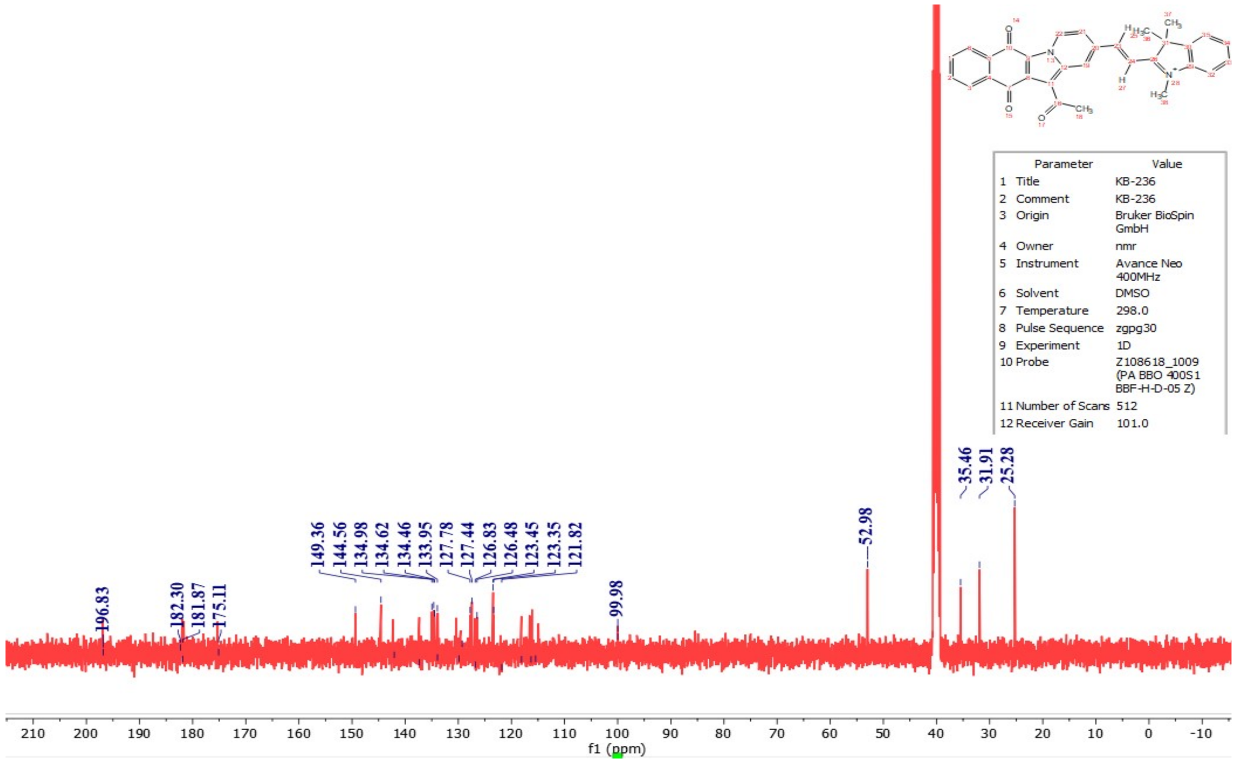

Parameter Value
1 Title KB-236
2 Comment KB-236
3 Origin Bruker BioSpin GmbH
4 Owner nmr
5 Instrument Avance Neo 400MHz
6 Solvent DMSO
7 Temperature 298.0
8 Pulse Sequence zgpg30
9 Experiment 1D
10 Probe Z108618_1009 (PA BBO 400S1 BBF-H-D-05 Z)
11 Number of Scans 512
12 Receiver Gain 101.0
196.83
182.30
181.87
175.11
149.36
144.56
134.98
134.62
134.46
133.95
127.78
127.44
126.83
126.48
123.45
123.35
121.82
99.98
52.98
35.46
31.91
25.28
f1 (ppm)

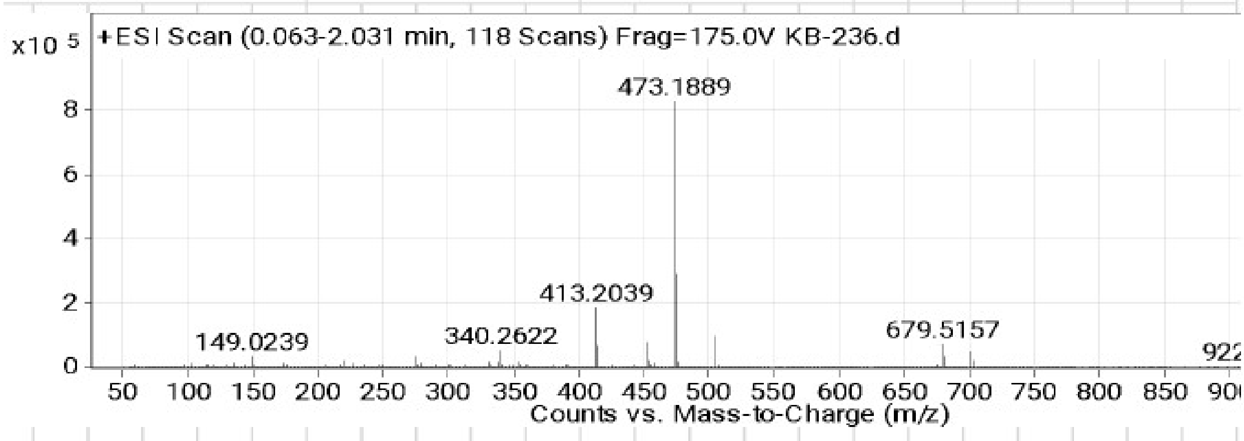

x10 5
+ESI Scan (0.063-2.031 min, 118 Scans) Frag=175.0V KB-236.d
473.1889
413.2039
149.0239
340.2622
679.5157
922
Counts vs. Mass-to-Charge (m/z)

UV-visible absorption and fluorescence spectra of ADTI in different solvents

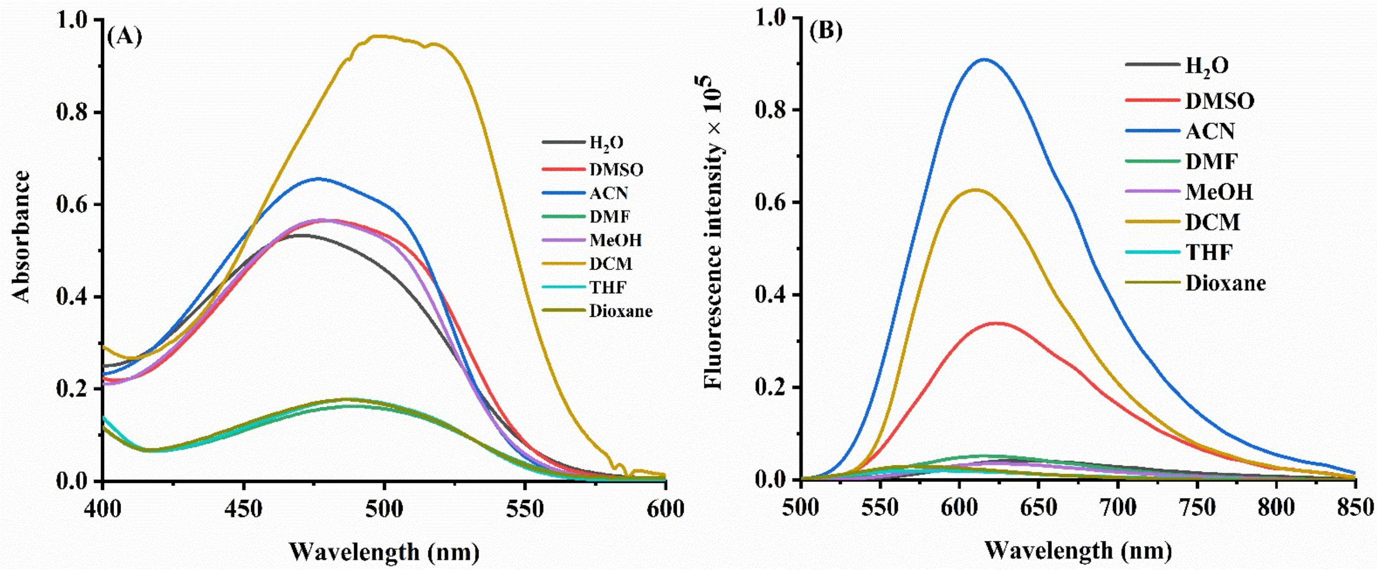

Figure S 1: (A) UV-vis absorption; **(B)** Emission spectra of probe in different solvents

Lippert Mataga plot

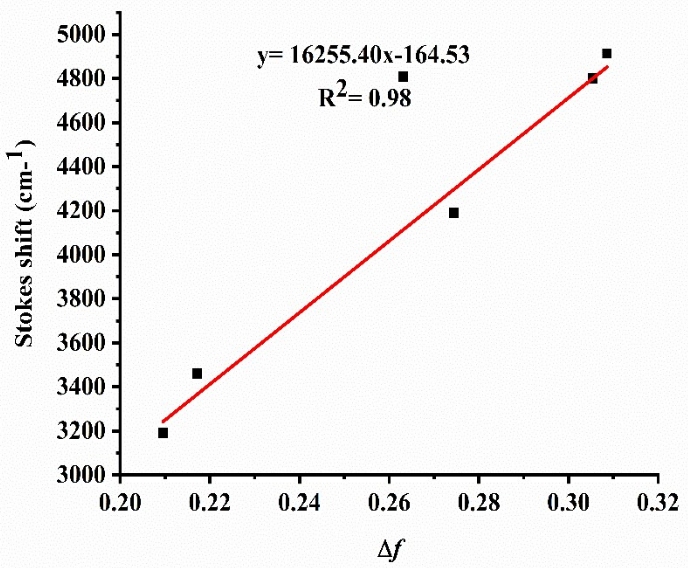

Figure S 2: Lippert Mataga plot

Visible changes in probe solution under naked eye and UV-lamp in the presence of analytes

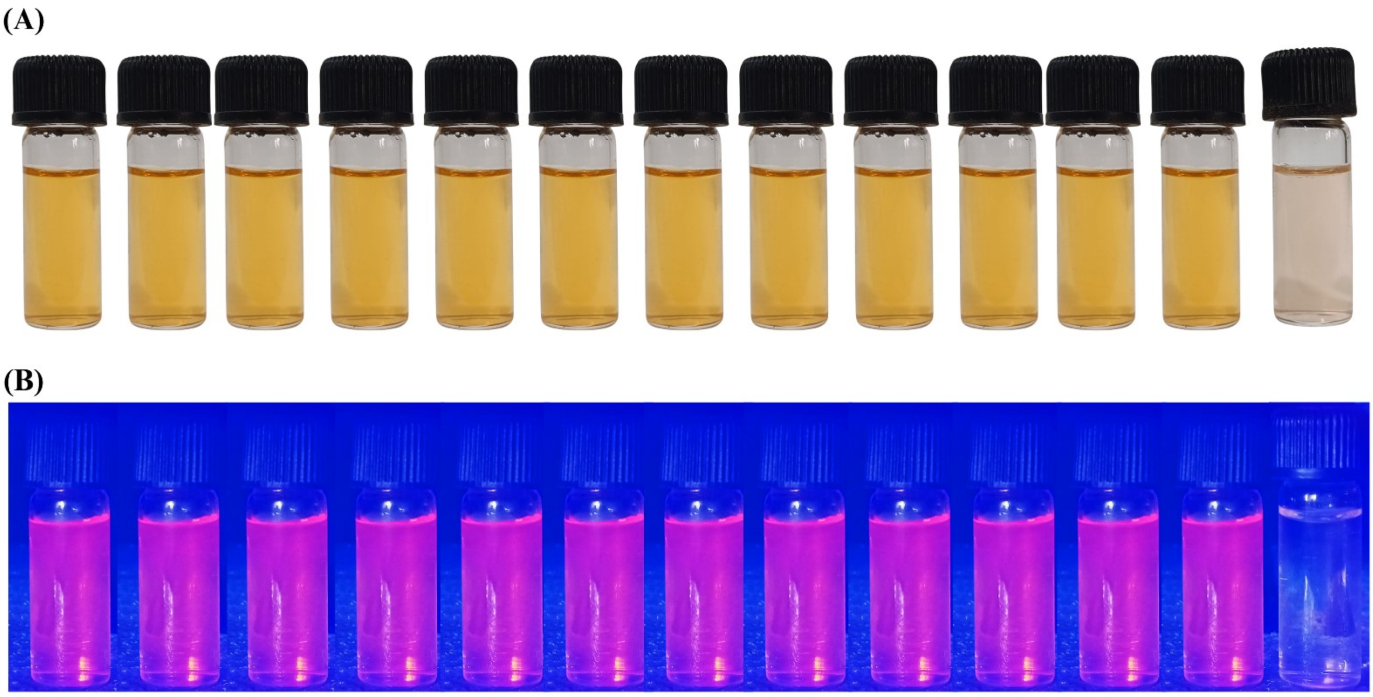

Figure S 3: (A) Visible changes in the color of **ADTI** in the presence of anions and (**B**) Under UV lamp

Photophysical attributes of ADTI

Table S 1: Photophysical attributes of probe in different solvent

Solvents	Absorbance (nm)	Wavenumber (cm^{-1})	Fluorescence (nm)	Wavenumber (cm^{-1})	Stokes shift (cm^{-1})
**H_2O**	471.0	21231.4	641.0	15600.6	5630.8
**DMSO**	480.0	20833.3	624.0	16025.6	4807.7
**CH_3CN**	476.0	21008.4	617.0	16207.4	4800.9
**DMF**	489.0	20449.9	615.0	16260.2	4189.7
**MeOH**	477.0	20964.4	623.0	16051.4	4912.9
**DCM**	503.0	19880.7	609.0	16420.4	3460.3
**THF**	488.0	20491.8	578.0	17301.0	3190.7
**1,4-Dioxane**	487.0	20533.9	572.0	17482.5	3051.4

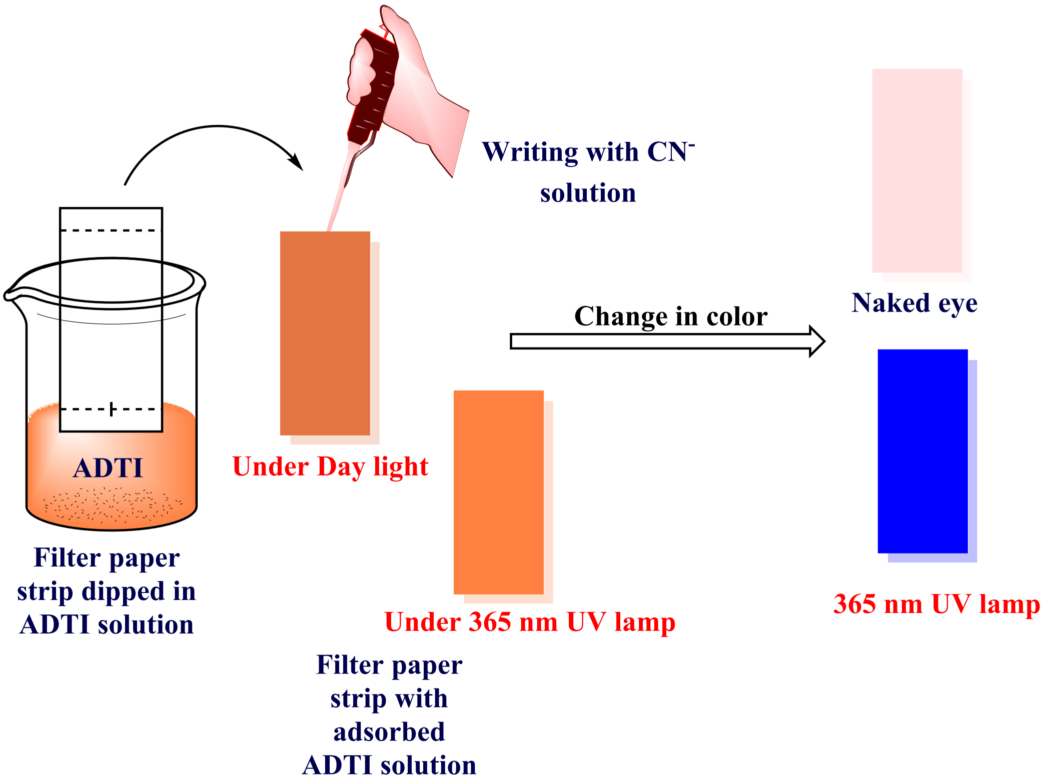

Figure S 4: Pictorial representation of color change on addition of cyanide ion to **ADTI** loaded filter paper strip

Table S 2: Truth table for INHIBIT logic gate

Input 1 (**ADTI**)	Input 2 (CN^-)	Output (λ_{emi} 641 nm)
1	0	1
1	1	0
0	1	0
0	0	0

Comparison of developed probe with previously reported probe

Table S 3: Comparison of the developed sensor **ADTI** with previously reported sensor

**Sr. No.**	**Authors group**	**Structure**	**λ_{emi}**	**Sensing medium**	**LOD**	**References**
**1**	**Park *et al.* *(2020)***	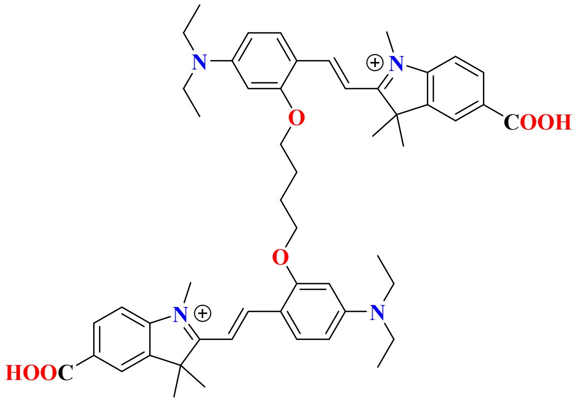	**584 nm**	**H_2O**	**1.53* 10^{-8}M**	[1]

**2**	**Li *et al. (2019)***	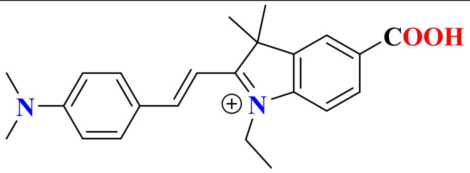	**603 nm**	**H_2O**	**3.34 × 10^{-7} M**	[2]
**3**	**Kim *et al. (2017)***	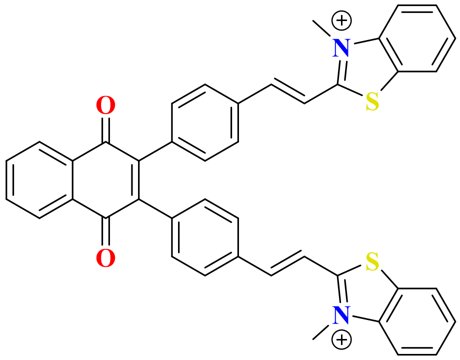	**566 nm**	**80% (DMF/H_2O)**	**4.97* 10^{-7} M**	[3]
**4**	**Li *et al. (2024)***	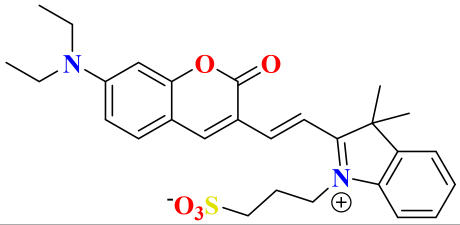	**495nm**	**DMSO/PBS**	**0.81 µM**	[4]
**5**	**Kiran *et al. (2024)***	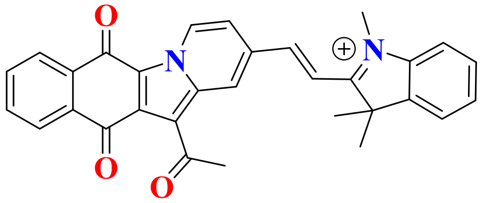	**641 nm**	**H_2O**	**3.78 nM**	**This work**

References

[1] J.H. Park, R. Manivannan, P. Jayasudha, Y.-A. Son, Selective detection of cyanide ion in 100 % water by indolium based dual reactive binding site optical sensor, J. Photochem. Photobiol. A Chem. 397 (2020) 112571. https://doi.org/https://doi.org/10.1016/j.jphotochem.2020.112571.

[2] J. Li, Z. Chang, X. Pan, W. Dong, A.Q. Jia, A novel colorimetric and fluorescent probe based on indolium salt for detection of cyanide in 100% aqueous solution, Dye. Pigment. 168 (2019) 175–179. https://doi.org/10.1016/j.dyepig.2019.04.059.

[3] I.J. Kim, M. Ramalingam, Y.A. Son, A reaction based colorimetric chemosensor for the detection of cyanide ion in aqueous solution, Sensors Actuators, B Chem. 246 (2017) 319–326. https://doi.org/10.1016/j.snb.2017.02.015.

[4] D. Li, S. Peng, X. Zhou, L. Shen, X. Yang, H. Xu, C. Redshaw, C. Zhang, Q. Zhang, A coumarin–hemicyanine deep red dye with a large stokes shift for the fluorescence detection and naked-eye recognition of cyanide, Molecules. 29 (2024). https://doi.org/10.3390/molecules29030618.
